# Effects of Transcutaneous Auricular Vagus Nerve Stimulation on Posttraumatic Stress Disorder Symptoms in World Trade Center Responders: A Feasibility and Acceptability Study

**DOI:** 10.3390/ijerph23030401

**Published:** 2026-03-21

**Authors:** Shubham Debnath, Haley M. Cook, Pooja Shaam, Laura Ryniker, Fylaktis Fylaktou, Lynne Lieberman, Molly McCann Pineo, Kristina M. Deligiannidis, Theodoros P. Zanos, Rebecca M. Schwartz

**Affiliations:** 1 Northwell, New Hyde Park, NY 11040, USA; sdebnath@northwell.edu (S.D.); hcook1@northwell.edu (H.M.C.); pshaam@northwell.edu (P.S.); lryniker@northwell.edu (L.R.); ffylaktou@northwell.edu (F.F.); llieberman1@northwell.edu (L.L.); mmccann1@northwell.edu (M.M.P.); kdeligian1@northwell.edu (K.M.D.); 2 Feinstein Institutes for Medical Research, Northwell, Manhasset, NY 11030, USA; 3 Department of Psychiatry, Zucker School of Medicine at Hofstra/Northwell, Hempstead, NY 11549, USA; 4 Department of Occupational Medicine, Epidemiology and Prevention, Northwell, Great Neck, NY 11021, USA; 5 Department of Emergency Medicine, Zucker School of Medicine at Hofstra/Northwell, Hempstead, NY 11549, USA; 6 Department of Medicine, Zucker School of Medicine at Hofstra/Northwell, Manhasset, NY 11030, USA

**Keywords:** transcutaneous auricular vagus nerve stimulation (taVNS), posttraumatic stress disorder (PTSD), World Trade Center (WTC) responders, feasibility, acceptability

## Abstract

**Highlights:**

**Public health relevance—How does this work relate to a public health issue?**

- This study addresses the persistent PTSD treatment gap among 9/11 World Trade Center responders by testing a home-based, non-invasive intervention to reduce barriers to engagement.
- This randomized, double-blind trial evaluates taVNS specifically in clinician-confirmed PTSD, advancing neuromodulation beyond prior work and into a high-need public health cohort.

**Public health significance—Why is this work of significance to public health?**

- The trial demonstrates strong feasibility in a real-world responder population, with 90.6% retention and an average adherence of 80.23% during eight weeks of daily at-home use.
- The intervention highlights targeted clinical signals, most notably a significant improvement in CAPS-5 Cognition/Mood and ≥10-point CAPS-5 reductions in 40% of the active group, indicating potential symptom relief in domains tied to day-to-day functioning and quality of life.

**Public health implications—What are the key implications or messages for practitioners, policy makers and/or researchers in public health?**

- Public health programs, such as the WTCHP, can pilot taVNS as a low-burden adjunct to evidence-based psychotherapies to improve treatment reach and adherence among trauma-exposed populations.
- Health systems can incorporate taVNS into stepped-care pathways as an acceptable, minimally risky option for patients unwilling or unable to engage in intensive psychotherapy, potentially increasing overall PTSD treatment uptake and reducing unmet need.

**Abstract:**

Background: Responders to the September 11, 2001, WTC attacks experience high rates of PTSD, and existing treatments often lead to high dropout and low care use. Objectives: This randomized, double-blind, sham-controlled trial assesses the feasibility and acceptability of transcutaneous auricular vagus nerve stimulation (taVNS) as a potential PTSD treatment for 9/11 responders. Methods: A total of 32 WTC responders aged 18+ with PTSD, recruited via the World Trade Center Health Program, participated; those with current psychosis, unstable medical conditions, or recent trial involvement were excluded. Participants were randomly assigned to taVNS or sham groups and asked to use the device for 15 min daily for 8 weeks, with staff and participants blinded. Primary outcomes included recruitment, adherence, retention, and feedback. Secondary outcomes examined changes in depression (PHQ-9), anxiety (GAD-7), and sleep (PSQI). Data were analyzed with mixed-effects models focusing on PTSD and mental health symptoms. Results: The taVNS group showed modest PTSD improvement, with a 10-point CAPS-5 reduction in 40% of stimulation participants versus 28.5% sham; no significant differences in self-reported symptoms were found. Discussion: Daily taVNS over eight weeks is feasible and acceptable, warranting larger studies to detect differences and identify subgroups with greater benefit. Trial registration: “taVNS to Reduce PTSD Symptoms in WTC Responders” (NCT05212714); registered 9 September 2021.

## 1. Introduction

The terrorist attacks on 11 September 2001 exerted profound and enduring impacts on the responders who delivered emergency services at Ground Zero. An estimated 40,000 to 60,000 individuals participated in rescue and recovery efforts, thereby exposing them to unprecedented traumatic incidents [1]. Post-traumatic stress disorder (PTSD) remains the most common mental health diagnosis among these responders, with prevalence rates exceeding those observed in the general population [2]. Research indicates that symptoms of PTSD, along with comorbid conditions such as depression and anxiety, may adopt a chronic trajectory among World Trade Center (WTC) responders [3]. The persistent nature of these symptoms amplifies the risk of reemergence of PTSD symptoms, regardless of prior remission, upon exposure to subsequent traumatic events [4].

Despite the availability of evidence-based treatments (EBTs) for PTSD, such as Prolonged Exposure (PE), Cognitive Processing Therapy (CPT), and Eye Movement Desensitization and Reprocessing (EMDR), these approaches are associated with high dropout rates of approximately 30%, driven by factors such as stigma and the emotionally demanding nature of the interventions [5]. Psychopharmaceutical approaches demonstrate varying efficacy without psychotherapy treatments [6]. Additionally, many treatment protocols require significant time commitments, which can be challenging for clients struggling with PTSD marked by avoidance [7].

The World Trade Center Health Program (WTCHP) provides free mental health treatment specifically for conditions associated with rescue, recovery, and clean-up efforts. However, a 2017 report indicated that only 40% of WTC responders and 20% of Fire Department of New York (FDNY) responders certified for mental health treatment received care through the program [8]. Low utilization rates may reflect a range of barriers, including limited availability to utilize services, lack of awareness of existing programs, concerns about treatment efficacy, stigma related to seeking mental health care, and the perceived absence of acceptable treatment alternatives. These statistics emphasize the need for more effective and acceptable treatment options for responders.

Neurostimulation techniques, such as vagus nerve stimulation (VNS), are utilized to address various mental health disorders with varying levels of invasiveness and potential side effects [9]. VNS has demonstrated effectiveness in treating diseases like epilepsy, depression, headache disorders, rheumatoid arthritis, and Crohn’s disease [10]. VNS potentially benefits psychiatric disorders by downregulating stress response activity in the brain [11]. Studies indicate that VNS enhances memory consolidation and prevents the reinstatement of conditioned fear, suggesting promise as an additional therapy for PTSD [12]. Recent human studies demonstrate significant impacts of VNS in facilitating fear extinction and modulating stress responses, indicating its potential efficacy and safety for addressing PTSD symptoms [13]. Porges’ Polyvagal Theory further contextualizes these findings, as it identifies the ventral vagal complex as a key mediator of social engagement and threat response regulation [14]. Given that these processes are disrupted in PTSD, taVNS may exert therapeutic effects in part by enhancing ventral vagal activity through stimulation of the auricular branch of the vagus nerve [14].

Transcutaneous Auricular VNS (taVNS) has emerged as a promising alternative for treating PTSD [15]. Research indicates that the tragus, concha, and cymba concha areas of the outer ear are unique in having direct access to the vagus nerve at the skin’s surface [16]. This anatomical feature enables non-invasive approaches like taVNS, which send electrical impulses directly to the vagus nerve through the cymba concha of the ear [17]. This specific modality of taVNS has undergone evaluation in individuals diagnosed with major depressive disorder with peripartum onset and rheumatoid arthritis, demonstrating significant impacts on cerebral blood flow and enhanced resting-state functional connectivity in the cingulate cortex and insula, regions of the brain integral to the interpretation of interoceptive signals and the processing of pain [18,19,20]. Previous studies demonstrate that taVNS can modulate the activity of mood-related brain centers [21], reduce inflammation [22], and enhance the consolidation of extinction memories [12], all of which are beneficial in reducing PTSD symptoms. However, to date, taVNS has not been evaluated in people living with PTSD.

The primary aim of this study was to assess the feasibility of daily taVNS among 9/11 responders with PTSD over eight weeks, focused on recruitment rates, intervention adherence, and retention. The secondary aim evaluated acceptability by examining questionnaire completion times, data quality, biological data collection, refusal rates, and satisfaction scores. Additionally, an exploratory aim investigated the potential efficacy of taVNS versus sham stimulation in reducing PTSD, depression, and anxiety symptoms. This study leveraged the latest advances in non-invasive bioelectronic medicine technology and applied them to a population with significant mental health needs. The novelty of this work lies in its potential to offer a new therapeutic approach that is both accessible and effective for those whose symptoms have been insufficiently addressed by traditional treatments.

## 2. Materials and Methods

### 2.1. Study Design

This pilot study used a randomized, double-blind, placebo-controlled, parallel design with 2:1 allocation to taVNS or sham. The taVNS group received stimulation from a handheld device delivering electrical impulses to the auricular branch of the vagus nerve; the sham group used an identical device without active stimulation. The trial was conducted at a major research institution from February 2022 to January 2024 and was approved by the Institutional Review Board (IRB), with written informed consent obtained from all participants. The study was registered on ClinicalTrials.gov (NCT05212714) registered 9 September 2021, adhered to CONSORT guidelines, and participants were compensated for time and travel. No changes to trial methods, outcomes, or analyses were made after commencement. No interim analyses or formal stopping guidelines were planned. As an exploratory pilot focused on feasibility and acceptability, the target sample size (*n* = 32; 2:1 allocation) was determined pragmatically based on available resources and anticipated recruitment.

### 2.2. Study Participants and Recruitment

Researchers recruited 32 participants from a pool of 2536 from a large health monitoring program for WTC responders who consented to being contacted for research purposes and had been diagnosed with PTSD. To determine eligibility, researchers identified individuals who scored a 44 or higher on the Posttraumatic Stress Disorder Checklist for DSM-5 (PCL-5) during annual monitoring visits between 2018 and 2020 [23], following Veterans Affairs screening guidelines for probable PTSD [24].

Final inclusion criteria for participation included: 1) age 18 or older; 2) meeting diagnostic criteria for current PTSD as assessed by the Clinician-Administered PTSD Scale for DSM-5 (CAPS-5); and 3) willingness to participate in the study and provide informed consent in English [23].

Exclusion criteria included: 1) active psychosis, mania, or substance dependence as indicated by the Mini International Neuropsychiatric Interview (M.I.N.I. [25]); 2) current suicidal or homicidal intent or plan assessed by the Columbia-Suicide Severity Rating Scale (C-SSRS; [26]; 3) medical conditions that may impact safety or results, including psychotropic medications, cardiovascular or neurological conditions, most recent cancer diagnoses, or active ear disease; 4) refusal to remove certain ear piercings; 5) history of vagotomy; 6) pregnancy; 7) the presence of implanted medical devices, such as pacemakers or devices interfering with study equipment; and 8) recent participation in another trial. The extensive exclusion criteria in this study are essential to ensure participant safety, enhance the validity and reliability of the findings, and maintain ethical standards by enrolling a homogeneous group diagnosed with PTSD without confounding psychiatric conditions, thus optimizing the efficacy assessment of taVNS.

### 2.3. Randomization and Blinding

Eligible patients were randomly assigned to the taVNS or sham group in a 2:1 ratio. The research staff, in accordance with IRB approval, used the Biostatistics Randomization Management System—an internal platform that automatically allocates participants to the treatment or sham group based on established arms and stratification parameters. Those responsible for enrolling participants did not have access to the random allocation sequence; the assignment was carried out by the system and disclosed only to the unblinded device programmer, who played no role in assessing outcomes. Participants were kept unaware of the stimulation conditions, while primary investigators and researchers remained blinded to the taVNS conditions.

### 2.4. Procedure

Eligible participants provided consent and were fitted with a custom taVNS device, a wearable Transcutaneous Electrical Nerve Stimulation (TENS) unit called the Nēsos Stim100 System, developed by Nēsos Corp. in Redwood City, California. The system delivers electrical stimulation to the auricular branch of the vagus nerve via the cymba concha. Classified as a non-significant risk device, it does not require an Investigational Device Exemption (IDE), facilitating its use under minimal regulatory constraints.

The STIM100 System comprises several integral components. The external stimulator is made of acrylonitrile–butadiene–styrene (ABS) plastic, selected for its documented safety in medical applications. It generates electrical pulses that are delivered transcutaneously via a neural interface earpiece worn on each ear. Each earpiece contains two electrodes constructed from medical-grade, biocompatible conductive hydrogel or silicone. The earpiece itself is composed of silicone, a material with established safety in wearable devices, ensuring reliable contact with the user’s skin.

The external stimulator is a handheld device connected via cables to both earbuds and delivers charge-balanced, current-controlled, biphasic square-wave pulses at 20 kHz with a pulse width of 20 µs. The earbuds are individually molded to fit each participant’s ear, ensuring proper electrode contact. Before placement in the ear, a conductive electrolyte solution, such as Signa spray, is applied to reduce resistance at the electrode–skin interface.

A proprietary smartphone application from Nēsos manages device functionality, allowing users to switch stimulation on and off, monitor electrode-to-skin contact through periodic impedance measurements, and receive notifications for poor contact and session timing. The STIM100 System was developed and tested in accordance with ISO 13485:2016 to ensure compliance with applicable regulatory clauses [27]. Although its use in this study is investigational, it is considered minimal risk. The FDA considers it so because the device is non-implantable, non-life-sustaining, and not essential for disease diagnosis, mitigation, or treatment, thereby posing no significant threat to the health, safety, or welfare of subjects.

In the initial session, the stimulation amplitude for each participant was set at 75% of their perceptual threshold, ranging from 2.0 to 4.0 mA. This accommodated individual differences in the electrode–skin interface and anatomy. A step-up and step-down binary parametric search was used to establish these thresholds and stimulation parameters. Participants received ten-second stimulation trains and were asked whether they felt any sensation. Adjustments of 50% increases or decreases in intensity followed affirmative or negative responses, respectively, allowing determination of the minimum threshold. This was typically experienced as a “tickle” or “pricking” sensation, indicating effective vagus nerve stimulation at both ear sites. After the perceptual threshold was established, the stimulation amplitude was set for participants in the stimulation group, while the current was set to 0.0 mA for participants in the sham group.

Overall, the STIM100 System provided a controlled and secure means for delivering taVNS, ensuring regulatory compliance while permitting individualized adjustments to optimize the intervention across a diverse participant population.

After customization and testing of the devices, participants attended a baseline visit to ensure proper fit and calibrate the stimulation level. During this visit, participants were provided with the taVNS device and a locked-down phone to interface with the device and record usage data. They were also given baseline self-report surveys to complete. Participants were trained to use the device and completed their first 15-minute session under the supervision of research staff. Participants in the study received financial incentives ranging from $20 to $35, along with a consistent travel reimbursement of $25 per participant for the CAPS screening interviews, taVNS device fitting, baseline measurements, and follow-up measurements, all paid via an Amazon gift card at the end of each study visit.

Participants in both the taVNS and sham groups were instructed to self-administer 15 min at-home treatment sessions daily for 8 weeks. Adverse events were monitored systematically at each contact and at the 8-week follow-up via participant self-report, device logs, and staff queries; any adverse events were recorded and assessed for relatedness and severity in accordance with IRB-approved procedures. The devices included a safety feature that prevented use beyond the prescribed daily time points, and the stimulation parameters were set by the investigator and could not be altered by the participants. Each use was time-stamped and recorded by the application, which tracked compliance without capturing personal information. All phones with the STIM100 application were collected at the end of the study. Participants returned 8 weeks post-baseline for a follow-up visit, during which the same self-report measures were repeated.

### 2.5. Measures

Demographic information, including age, gender, race, ethnicity, and education level, was collected, along with data on past and current mental health treatments, such as psychotherapy sessions, treatment duration, and medications used. The primary outcome measure was feasibility, assessed by monthly recruitment rates, adherence to the taVNS intervention, eight-week retention rate, and completion rates of study assessments. Adherence was operationalized as the percentage of completed stimulation sessions out of 56 possible sessions (one per day over eight weeks), as recorded by time-stamped device logs.

Acceptability served as the secondary outcome measure and was assessed by recording the time and completion rates for questionnaires and biological assessments, the percentage of missing questionnaire data, refusal rates for biological procedures, and scores on the taVNS Satisfaction and Usefulness Questionnaire to inform future research [28].

Exploratory outcomes included changes in PTSD symptoms using the Clinician-Administered PTSD Scale for DSM-5 (CAPS-5; [23]) and the PTSD Checklist for DSM-5 (PCL-5; [29]). Overall scores and clinically significant decreases (>10) were assessed. Anxiety symptoms were assessed using the Generalized Anxiety Disorder 7-item scale (GAD-7; [30]), and depression symptoms were evaluated with the Patient Health Questionnaire-9 (PHQ-9; [31]). Sleep quality was assessed using the Pittsburgh Sleep Quality Index (PSQI; [32]), with assessments conducted both at baseline and at an 8-week follow-up.

### 2.6. Data Analysis

Descriptive statistics, specifically frequencies, were utilized to assess feasibility outcomes, including monthly recruitment rates, adherence to the taVNS intervention, 8-week retention rate, and completion rates of study assessments. Acceptability was evaluated using metrics on time and completeness for questionnaire and biological data collection, refusal rates, and satisfaction scores. Although the study was not powered for efficacy, exploratory analyses examined changes in PTSD symptoms using CAPS-5 severity scores and PCL-5 self-report scores, both in terms of overall changes and clinically meaningful changes of > 10 points. Analysis of variance (ANOVA) was conducted to compare baseline and follow-up scores between the treatment and control groups. This approach enabled identification of significant differences in CAPS-5 total scores and symptom clusters (intrusion, avoidance, cognition/mood, arousal/reactivity), as well as in GAD-7, PHQ-9, and PSQI measures. Intention-to-treat analyses addressed potential attrition, ensuring a comprehensive assessment of study outcomes. Missing data handling was not prespecified; analyses used available-case data. Demographic data were collected, including age, gender, race, ethnicity, education level, and information on past and current mental health treatments.

## 3. Results

Recruitment ran from March 2022 to December 2023, and the 8-week follow-up was completed by January 2024. The trial ended as planned at the completion of the recruitment period and scheduled follow-ups. A total of forty-nine responders were screened for eligibility to participate in the study. Of these, thirteen (26.5%) were excluded because they did not meet the inclusion criteria. Additionally, three respondents (6.1%) were lost to follow-up before scheduling their fitting appointments, and one respondent (2.0%) was excluded because the enrollment period had closed.

Ultimately, thirty-two participants provided informed consent and were enrolled in the study between March 2022 and December 2023. During the study, three participants did not complete the intervention: one participant elected to withdraw from the study, another participant was withdrawn from the study by the investigator due to noncompliance with the treatment protocol, and the third participant was unable to adhere to the device usage requirements; however, they completed the 8-week follow-up questionnaires.

**Figure 1:**
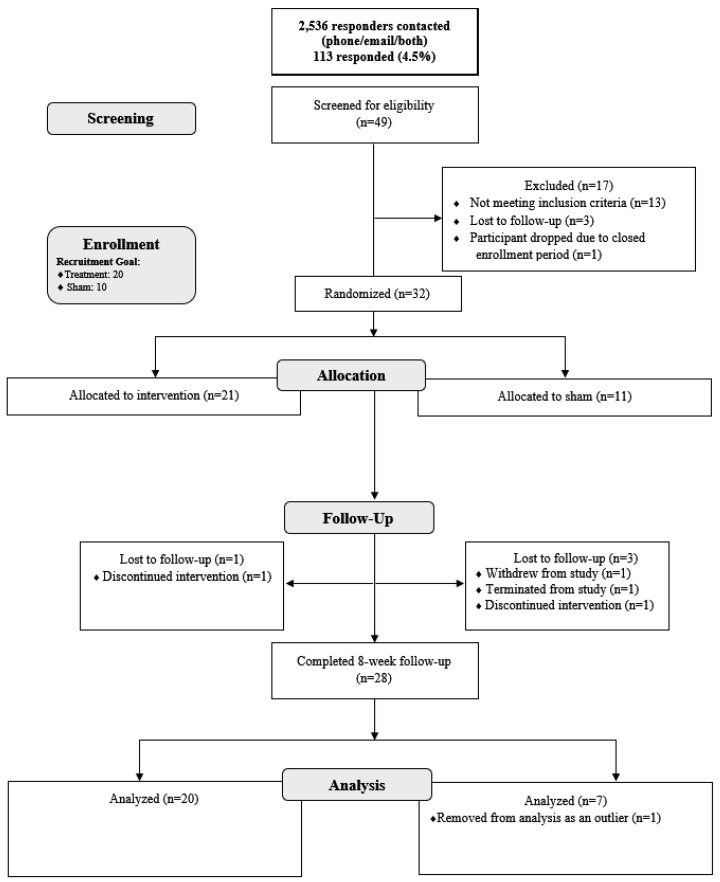
CONSORT Flow Diagram.

The final analysis included 32 participants who completed their baseline visit, of whom 29 completed the study as per protocol. The adherence rates and reasons for attrition are illustrated in the CONSORT flow diagram (Figure 1).

Participants in this study (*n* = 32) were WTC responders who met diagnostic criteria for current PTSD as assessed by the CAPS-5. Their ages ranged from 47 to 69 years (M = 60.35, SD = 6.2), and the majority were male (81%, *n* = 26). Most participants self-identified as White (78%, *n* = 25) and non-Hispanic (84.4%, *n* = 27). The sample was highly educated, with most having attended college (84.4%, *n* = 27) and over half (53%, *n* = 17) holding a college or postgraduate degree. Most participants (84.4%, *n* = 27) also had health insurance at baseline. Given the small sample size and pilot design, formal statistical comparisons of baseline demographic characteristics between groups were not conducted. However, a descriptive review of Table 1 indicates the groups were generally comparable on key variables, including sex, race, ethnicity, education, and prior mental health treatment.

**Figure 2:**
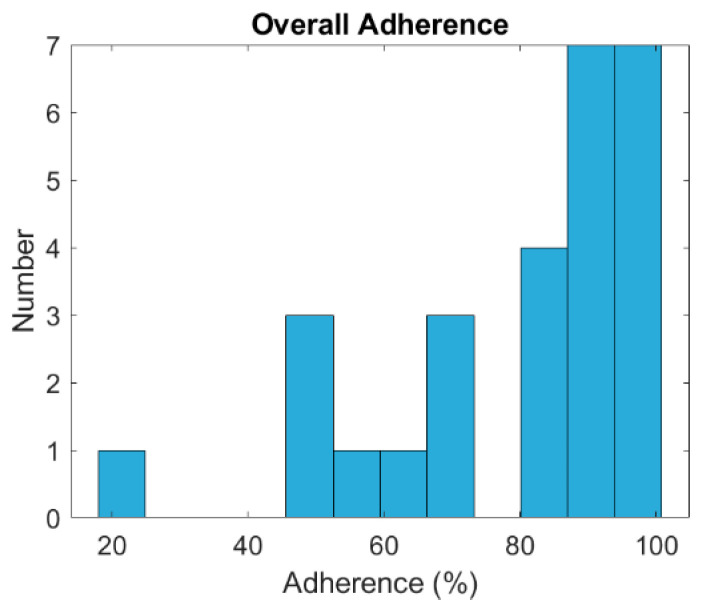
Overall adherence to the daily taVNS stimulation protocol.

Recruitment and adherence rates demonstrated adequate feasibility and acceptability within this sample. Researchers contacted 2536 WTC responders via email or by phone when email was unavailable, and 113 individuals expressed interest in participation. Among these, 49 respondents completed the CAPS inclusion interview, and 39 (79.6%) met the eligibility criteria. Investigators lost seven eligible individuals to follow-up or excluded them prior to enrollment, resulting in the enrollment of 32 participants (65.3% of those interviewed) who subsequently completed baseline assessments. Following additional exclusions, one due to unusable bloodwork and one for outlying PTSD scores, the final sample included 20 participants in the active stimulation group and seven in the sham group. The study achieved a retention rate of 90.6%, surpassing the predetermined target of 70%, with only three participants withdrawing or lost to follow-up. No serious adverse events occurred in either group. Minor, expected device-related sensations (e.g., transient ‘tickle’ or ‘pricking’) were reported during threshold setting; no device-related adverse events leading to discontinuation were observed. Participants maintained an average compliance rate of 80.23% with daily taVNS use over eight weeks, ranging from 17.86% to 100% (see Figure 2). Researchers obtained nearly complete data for self-report and biological measures, with minimal missing questionnaire responses and no refusals to provide blood samples. The taVNS Satisfaction and Usefulness Questionnaire reflected a high satisfaction rate of 98.5%.

**Figure 3:**
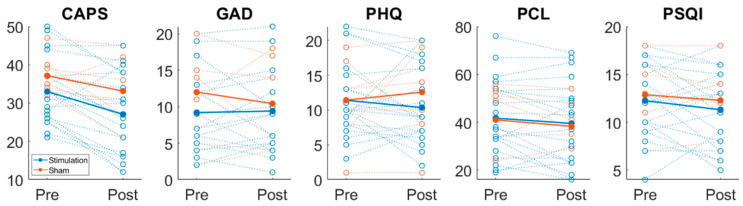
Changes in clinical scores from baseline to follow-up for all participants. Changes in CAPS-5, GAD-7, PHQ-9, PCL-5, and PSQI scores for all participants are shown. Each open circle trace represents a single individual, and the thicker, filled circles indicate the average change for each score. Traces are colored blue and orange for stimulation and sham groups, respectively.

Changes in symptoms from baseline to the 8-week follow-up were evaluated using the CAPS-5 and PCL-5 for PTSD, the PHQ-9 for symptoms of depression, GAD-7 for symptoms of anxiety, and the PSQI for sleep disturbances (see Table 2). These analyses were conducted with an exploratory focus because the study was not powered to assess efficacy on these measures. Figure 3 displays symptom scores at both time points. CAPS-5 scores decreased by an average of 5.95 points (*p* = 0.0608) in the active stimulation group, compared to a 4-point decrease (*p* = 0.3083) in the sham group; however, the difference between baseline and follow-up in both groups was not statistically significant. Clinically meaningful (≥ 10-point) decreases in CAPS-5 score were observed in 40% (*n* = 8) of the active group; when these participants were isolated, the average CAPS-5 score decreased from 34 to 22.25 (*p* = 0.043). Meanwhile, only 28.5% (*n* = 2) of the sham group had a CAPS-5 drop of at least 10 points, from an average of 36 to 24. These reductions in the sham group, however, are descriptive and cannot be evaluated for statistical significance due to the small sample sizes. Both the active and sham groups had non-significant changes in GAD-7, PHQ-9, and PCL-5 scores. The PSQI analysis revealed that 26 out of 27 participants reported sleep difficulties (PSQI > 5). On average, the stimulation group’s score decreased by 0.95, while the sham group’s score decreased by 0.5714; neither group showed statistically significant improvements in sleep hygiene. No clear association was observed between changes in CAPS-5 scores and adherence; the active stimulation group averaged 82.7% adherence, while the sham stimulation group averaged 72.29%. Although the majority of participants adhered appropriately and demonstrated reductions in CAPS-5 scores, some participants used the device for 50–60% of the study duration (i.e., approximately 28–34 of 56 sessions) and nonetheless experienced decreases in clinical scores.

Participants were asked to use the device for 15 min daily for 8 weeks. The adherence range was 17.86–100%, with an average of 80.23%. The mode was 91.07% (51 out of 56 possible stimulation sessions).

**Figure 4:**
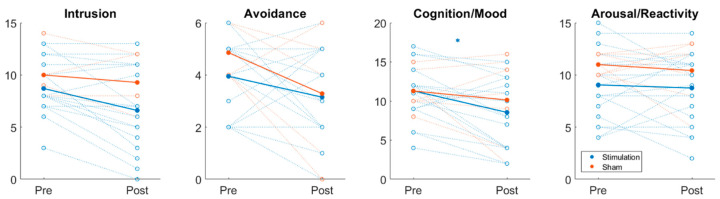
Changes in CAPS-5 subgroup scores from baseline to follow-up for all participants. Changes in CAPS-5, GAD-7, PHQ-9, PCL-5, and PSQI scores for all participants are shown. Each open circle trace represents a single individual, and the thicker, filled circles indicate the average change for each score. Traces are colored blue and orange for stimulation and sham groups, respectively.

Further analysis of the CAPS-5 subscales, as shown in Figure 4, revealed a statistically significant decrease in Cognition/Mood scores in the active stimulation group (M = 2.750, *p* = 0.0362), whereas the sham group showed a non-significant decrease (M = 1.143, *p* = 0.6237). Intrusion scores decreased in the active group (M = 2.100, *p* = 0.0527) but not in the sham group (M = 0.7143, *p* = 0.6370). Avoidance scores decreased in both groups, with a larger, though non-significant, decrease in the sham group (M = 1.5714, *p* = 0.0983) compared to the active group (M = 0.8000, *p* = 0.1113). Arousal/Reactivity scores showed minimal, non-significant changes in both groups.

CAPS-5 scores were also stratified by whether additional treatment occurred during the study period. Changes in CAPS-5 scores were categorized by stimulation and sham groups and by whether participants received any additional treatment during the 8-week study, including psychotherapy, medication, or inpatient hospitalization. Treatment had no effect on the active group, with average CAPS-5 scores decreasing from 33.1 to 26.3 and from 32.9 to 27.5 in participants without (*n* = 7) and with additional treatment (*n* = 13), respectively. In the sham group, however, the average CAPS-5 score of the untreated group (*n* = 5) decreased from 26 to 24, while the sham plus treatment group’s average score (*n* = 2) remained nearly unchanged, from 37.6 to 36.8. Still, these changes cannot be assessed for statistical significance due to the small sample sizes.

## 4. Discussion

This pilot study offers compelling preliminary data supporting the feasibility and acceptability of taVNS as a novel therapeutic modality for PTSD treatment in WTC responders. Despite inherent challenges in initial research engagement within this unique population, resulting in a modest final sample from a substantial initial pool, the high retention rate (90.6%) and robust adherence to the daily taVNS protocol (average 80.23%) underscore the intervention’s tolerability and perceived practicality. The active stimulation group also demonstrated higher average adherence (82.7%) than the sham group (72.29%), although this difference was not formally tested because of the small and unequal group sizes. Whether active stimulation influences participant engagement remains an important question for future trials to address. The generally positive participant feedback captured by the taVNS Satisfaction and Usefulness Questionnaire further bolsters the compelling case for the acceptability of this approach. While the observed reduction in overall CAPS-5 scores in the active stimulation group did not achieve statistical significance compared to the sham group, the clinically significant improvements manifested in a substantial subset of active participants (40% demonstrating a ≥ 10-point CAPS-5 reduction) strongly suggest the therapeutic potential of taVNS and warrant rigorous further investigation with larger samples. The absence of significant changes in secondary outcome measures, including symptoms of anxiety, depression, and sleep disturbance, indicates that taVNS may exert a more targeted effect on specific PTSD symptom clusters, particularly those related to cognition and mood, as evidenced by the significant improvement in the corresponding CAPS-5 subscale. This nuanced impact highlights the need for future research to delineate the precise mechanisms of action and optimize treatment protocols for broader efficacy.

The clinically significant improvements observed in a substantial subset of the active taVNS group (40% demonstrating a ≥ 10-point CAPS-5 reduction) suggest the potential for this modality to become a valuable addition to the current treatment landscape for PTSD. This is consistent with previous findings from studies employing VNS therapy, where significant, enduring reductions in CAPS-5 scores were noted, emphasizing neuromodulation’s promising role in addressing treatment-resistant PTSD [33]. Notably, these improvements were observed on the clinician-administered CAPS-5 but not on the self-report PCL-5. This pattern is consistent with prior literature indicating that clinician-rated and self-report PTSD measures do not always converge, potentially due to differences in the constructs assessed, the structured clinical probing inherent in the CAPS-5, or response biases associated with self-report instruments [34,35]. This divergence underscores the value of incorporating both clinician-administered and self-report measures in future trials to provide a more comprehensive assessment of treatment effects. This non-invasive approach shows promise for WTC responders and other trauma-exposed populations who may face barriers to engaging with traditional, more intensive therapies, potentially due to factors such as time commitment, emotional intensity, or accessibility [36]. While further research is needed to confirm these preliminary findings in larger samples and explore optimal treatment parameters, the magnitude of change observed in some individuals, coupled with the high adherence rates, warrants continued investigation into the clinical utility of taVNS for PTSD.

While VNS is approved and has been used as an alternative treatment for drug-resistant epilepsy, depression, and as a supplement to stroke recovery rehabilitation [37,38,39,40], non-invasive VNS methods have only been recently studied as a potential treatment for multiple conditions, including refractory epilepsy, depression, pre-diabetes, tinnitus, stroke, lupus, headache, and others [39,40,41,42,43]. Specifically for PTSD, transcutaneous cervical VNS (tcVNS) has been extensively tested, examining both clinical and physiological biomarkers, showing a reduction in sympathetic function measured by increased cardiac contractility (pre-ejection period amplitude) and increased photoplethysmogram amplitude, an increase in parasympathetic function measured by respiratory rate, and blocked stress-related increases in IL-6 [44,45,46]. However, there were no consistent significant changes in endpoints related to PTSD, depression, and anxiety clinical scores [44]. The results shown here are consistent with those from tcVNS studies, including significant improvements in cognition and mood. Polyvagal Theory provides additional context for these findings, as it designates the ventral vagal complex as a critical mediator of autonomic flexibility, social engagement, and threat-response regulation [14]. The observed enhancement in cognition and mood, but not arousal/reactivity, aligns with this framework, as the vagal afferent stimulation may selectively facilitate the restoration of ventral vagal pathways involved in emotional processing and safety detection, rather than the sympathetic circuits that underlie hypervigilance and the startle response [14].

The study’s limitations include a relatively small sample size, which precluded formal statistical comparisons of baseline demographic characteristics between groups and limited the power to detect between-group differences. The sample was primarily male (81%), White (78.1%), and non-Hispanic (84.4%), which may limit generalizability to other demographic groups, particularly female participants and individuals from diverse racial and ethnic backgrounds. The initial recruitment process posed challenges, and some participants were lost to follow-up for their 8-week post-therapy visit. Additionally, individuals experiencing a higher burden of mental health symptoms were more inclined to enroll, which may have introduced bias and affected the generalizability of the study’s findings. While the CAPS-5 is a clinician-administered diagnostic tool, the remaining measures relied on self-report, which may introduce response biases. Moreover, some overlap existed between study groups, as three of the six focus group participants subsequently enrolled in Aim 2 of this study; this should be considered when interpreting participants’ perspectives. All participants received stimulation below the sensory threshold at the same frequency; determining the optimal stimulation parameters for effectively targeting PTSD and whether these should be tailored to individuals or specific patient populations remains an important consideration. Lastly, while targeting the auricular branch of the vagus nerve remains a favorable approach for noninvasive stimulation, given its superficial location and accessibility via the cymba concha, further research is needed to confirm that vagal circuit engagement is sufficient to produce downstream effects.

Future research should explore the long-term effects of taVNS on PTSD symptoms and examine its efficacy in other trauma-exposed populations. These feasibility outcomes support the development of a fully powered, multi-site randomized controlled trial, with sample size informed by power analysis based on the effect sizes observed here, particularly for the CAPS-5 Cognition/Mood subscale. To strengthen recruitment and retention, future trials should expand outreach through community partnerships and engagement with patient advocacy groups, and offer flexible scheduling or remote participation options, while enrolling larger, more diverse samples. Objective outcome measures should be prioritized, including more frequent collection of heart rate variability and inflammatory biomarker data, either through whole blood processing or more frequent sampling, to clarify mechanisms of action and reduce reliance on self-report instruments. Such trials should also incorporate longer follow-up periods, stratified subgroup analyses, and formal baseline equivalence testing. Stimulation parameters should be systematically varied, including frequency, amplitude (sub- or suprasensory threshold), laterality, and regularity, to determine optimal protocols for individual participants or patient populations. However, because sensory thresholds can be easily affected by electrode impedance, placement, and skin conditions, future studies must ensure that these variations do not compromise blinding. Because no published study has examined taVNS in combination with concurrent pharmacotherapy for PTSD, the potential interaction between neuromodulation and psychotropic medications warrants investigation in future trials.

## 5. Conclusions

This study on the feasibility and acceptability of taVNS for treating PTSD in 9/11 WTC responders demonstrates promising preliminary results. Despite challenges in recruitment and sample sizes, retention and adherence rates were high, along with some improvements in PTSD symptoms, underscoring the potential of non-invasive VNS approaches as effective treatment. This study highlights the acceptability of this non-invasive and easy-to-use treatment as a valuable alternative or supplement to traditional therapies. Non-invasive VNS approaches hold potential to impact the therapeutic landscape for trauma-affected populations with unmet clinical needs, offering improved outcomes to individuals struggling with symptoms and effects of PTSD in their daily lives.

## Figures and Tables

**Table 1:** Demographic and Characteristics of Participants at Baseline, by Treatment and Control.

	Total (*n* = 32)		Treatment (*n* = 21)		Control (*n* = 11)	
Variable	*n*	*%*	*n*	*%*	*n*	*%*
Sex						
Female	6	18.8%	4	19.0%	2	18.2%
Male	26	81.3%	17	81.0%	9	81.8%
Latino/Hispanic						
Yes	5	15.6%	3	14.3%	2	18.2%
No	27	84.4%	18	85.7%	9	81.8%
Race ^a^						
White	25	78.1%	15	75.0%	10	90.9%
Asian	1	3.1%	1	5.0%		
Black/African American	4	12.5%	3	15.0%	1	9.1%
Native Hawaiian/Pacific Islander	1	3.1%	1	5.0%		
Highest Education						
Some high school	1	3.1%	1	4.8%		
High school graduate	3	9.4%	2	9.5%	1	9.1%
Post high school (not college)	1	3.1%	1	4.8%		
Some college	10	31.2%	7	33.3%	3	27.3%
Graduated from college	10	31.2%	6	28.6%	4	36.4%
Postgraduate	7	21.9%	4	19.0%	3	27.3%
Medical Insurance						
Yes	27	84.4%	17	81.0%	10	90.9%
No	5	15.6%	4	19.0%	1	9.1%
Currently Employed						
Yes	10	31.3%	7	33.3%	3	27.3%
No	22	68.8%	14	66.7%	8	72.7%
Retired						
Yes	19	59.4%	12	57.1%	7	63.6%
No	13	40.6%	9	42.9%	4	36.4%
Previous Mental Health Care Treatment						
Yes	29	90.6%	19	90.5%	10	90.9%
No	3	9.4%	2	9.5%	1	9.1%
Current Mental Health Treatment ^b^						
Yes	21	65.6%	12	63.2%	9	81.8%
No	9	28.1%	7	36.8%	2	18.2%

Note. ^a^ Missing: Race, Treatment group *n* = 1 (3.1%). ^b^ Missing: Current Mental Health Treatment, Treatment group *n* = 2 (6.3%).

**Table 2:** PTSD Symptom Severity of Participants at Baseline and Follow-Up.

	CAPS-5		GAD-7		PHQ-9		PCL-5		PSQI	
Baseline	M	SD	M	SD	M	SD	M	SD	M	SD
Treatment	33	8.69	9.2	5.33	11.4	5.31	41.7	16.1	12.25	3.85
Control	37.14	5.27	12	6.48	11.43	6.11	41	14.79	12.86	3.8
Follow-Up	M	SD	M	SD	M	SD	M	SD	M	SD
Treatment	27.05	10.68	9.4	5.36	10.35	5.29	39.5	17.63	11.3	3.96
Control	33.14	8.43	10.43	5.99	12.57	6.73	38.29	10.64	12.29	3.5

## Data Availability

The data that support the findings of this study are available from the co-corresponding authors upon reasonable request. The full trial protocol and prespecified data analysis plan are available on ClinicalTrials.gov (NCT05212714).

## Abbreviations

The following abbreviations are used in this manuscript:

9/11	11 September 2001
WTC	World Trade Center
PTSD	Posttraumatic Stress Disorder
EBT	Evidence-Based Treatment
PE	Prolonged Exposure
CPT	Cognitive Processing Therapy
EMDR	Eye Movement Desensitization and Reprocessing
FDNY	Fire Department of New York
VNS	Vagus Nerve Stimulation
taVNS	Transcutaneous Auricular Vagus Nerve Stimulation
tcVNS	Transcutaneous Cervical Vagus Nerve Stimulation
CAPS-5	Clinician-Administered PTSD Scale for DSM-5
PCL-5	PTSD Checklist for DSM-5
PHQ-9	Patient Health Questionnaire-9
GAD-7	Generalized Anxiety Disorder 7-item scale
PSQI	Pittsburgh Sleep Quality Index
IRB	Institutional Review Board
CONSORT	Consolidated Standards of Reporting Trials
IDE	Investigational Device Exemption
TENS	Transcutaneous Electrical Nerve Stimulation
FDA	U.S. Food and Drug Administration
ISO	International Organization for Standardization
M.I.N.I.	Mini-International Neuropsychiatric Interview
C-SSRS	Columbia-Suicide Severity Rating Scale
WTCHP	World Trade Center Health Program
ANOVA	Analysis of Variance
M	Mean
SD	Standard Deviation
fMRI	functional Magnetic Resonance Imaging
IL-6	Interleukin-6
